# Use of the Pfizer Respiratory Syncytial Virus Vaccine During Pregnancy for the Prevention of Respiratory Syncytial Virus–Associated Lower Respiratory Tract Disease in Infants: Recommendations of the Advisory Committee on Immunization Practices — United States, 2023

**DOI:** 10.15585/mmwr.mm7241e1

**Published:** 2023-10-13

**Authors:** Katherine E. Fleming-Dutra, Jefferson M. Jones, Lauren E. Roper, Mila M. Prill, Ismael R. Ortega-Sanchez, Danielle L. Moulia, Megan Wallace, Monica Godfrey, Karen R. Broder, Naomi K. Tepper, Oliver Brooks, Pablo J. Sánchez, Camille N. Kotton, Barbara E. Mahon, Sarah S. Long, Meredith L. McMorrow

**Affiliations:** ^1^Coronavirus and Other Respiratory Viruses Division, National Center for Immunization and Respiratory Diseases, CDC; ^2^Immunization Safety Office, National Center for Emerging and Zoonotic Infectious Diseases, CDC; ^3^Division of Reproductive Health, National Center for Chronic Disease Prevention and Health Promotion, CDC; ^4^Watts Healthcare Corporation, Los Angeles, California; ^5^The Research Institute at Nationwide Children’s Hospital, The Ohio State University College of Medicine, Columbus, Ohio; ^6^Harvard Medical School, Boston, Massachusetts; ^7^Drexel University College of Medicine, Philadelphia, Pennsylvania.

**Summary**

**What is already known about this topic?**

Nirsevimab is recommended in infants to prevent respiratory syncytial virus (RSV)-associated lower respiratory tract infection (LRTI). In August 2023, the Food and Drug Administration approved Pfizer RSV vaccine for pregnant persons at 32–36 weeks’ gestation to prevent RSV-associated LRTI in infants aged <6 months.

**What is added by this report?**

On September 22, 2023, CDC’s Advisory Committee on Immunization Practices recommended RSV vaccine for pregnant persons at 32–36 weeks’ gestation using seasonal administration (meaning September–January in most of the United States) to prevent RSV-associated LRTI in infants aged <6 months.

**What are the implications for public health practice?**

CDC recommends protecting all infants against RSV-associated LRTI through use of either the maternal RSV vaccine or infant receipt of nirsevimab.

## Abstract

Respiratory syncytial virus (RSV) is the leading cause of hospitalization among U.S. infants. Nirsevimab (Bevfortus, Sanofi and AstraZeneca) is recommended to prevent RSV-associated lower respiratory tract infection (LRTI) in infants. In August 2023, the Food and Drug Administration (FDA) approved RSVpreF vaccine (Abrysvo, Pfizer Inc.) for pregnant persons as a single dose during 32–36 completed gestational weeks (i.e., 32 weeks and zero days’ through 36 weeks and 6 days’ gestation) to prevent RSV-associated lower respiratory tract disease in infants aged <6 months. Since October 2021, CDC’s Advisory Committee on Immunization Practices (ACIP) RSV Vaccines Pediatric/Maternal Work Group has reviewed RSV epidemiology and evidence regarding safety, efficacy, and potential economic impact of pediatric and maternal RSV prevention products, including RSVpreF vaccine. On September 22, 2023, ACIP and CDC recommended RSVpreF vaccine using seasonal administration (i.e., during September through end of January in most of the continental United States) for pregnant persons as a one-time dose at 32–36 weeks’ gestation for prevention of RSV-associated LRTI in infants aged <6 months. Either maternal RSVpreF vaccination during pregnancy or nirsevimab administration to the infant is recommended to prevent RSV-associated LRTI among infants, but both are not needed for most infants. All infants should be protected against RSV-associated LRTI through use of one of these products.

## Introduction

In August 2023, the Food and Drug Administration (FDA) approved RSVpreF vaccine (Abrysvo, Pfizer Inc.) for pregnant persons to prevent RSV-associated lower respiratory tract disease and severe lower respiratory tract disease in infants aged <6 months (*1*,*2*). The Pfizer bivalent RSVpreF vaccine, which is the same formulation and dose approved for use in adults aged ≥60 years, contains stabilized prefusion F glycoproteins from RSV A and RSV B and is approved as a single 0.5 mL intramuscular dose administered during 32 through 36 weeks’ gestation.

In clinical trials among pregnant persons at 24–36 weeks’ gestation, more preterm births (<37 weeks’ gestation) were observed among RSVpreF vaccine recipients than placebo recipients, although the differences were not statistically significant (*1*,*2*). Available data were insufficient to establish or exclude a causal relationship between preterm birth and RSVpreF vaccine. FDA labeled the potential risk for preterm birth as a warning and approved RSVpreF vaccine for use in pregnant persons at 32–36 weeks’ gestation to avoid the potential risk for preterm birth at <32 weeks’ gestation, which is associated with increased risk for morbidity and mortality (*2*). More hypertensive disorders of pregnancy were observed among RSVpreF vaccine recipients compared with placebo recipients, although the differences were not statistically significant. FDA determined that, when RSVpreF is administered during 32–36 weeks’ gestation, the benefit of vaccination in preventing RSV-associated LRTI in infants outweighed risks, including the potential risk for preterm birth and hypertensive disorders of pregnancy (*1**,**2*).

On August 3, 2023, CDC’s Advisory Committee on Immunization Practices (ACIP) and CDC recommended nirsevimab (Beyfortus, Sanofi and AstraZeneca), a long-acting monoclonal antibody for prevention of severe RSV disease, for infants aged <8 months who are born during or entering their first RSV season and for children aged 8–19 months at increased risk for severe RSV disease entering their second RSV season (*3*). On September 22, 2023, ACIP and CDC recommended RSVpreF vaccine for pregnant persons as a one-time dose during 32–36 completed weeks’ gestation using seasonal administration (September–January in most of the continental United States) to prevent RSV-associated lower respiratory tract infection (LRTI) in infants. Either maternal RSVpreF vaccination during pregnancy or nirsevimab administration to the infant is recommended to prevent RSV-associated LRTI in infants, but both are not needed for most infants. This report describes new recommendations for the use of maternal RSVpreF during pregnancy and updated clinical guidance regarding the use of nirsevimab and maternal RSVpreF vaccine. These recommendations will be updated as new evidence becomes available. 

### Epidemiology of RSV in U.S. Infants

RSV is a common cause of LRTI in U.S. infants, most of whom are infected with RSV during the first year of life (*4*,*5*). All infants are at risk for experiencing severe RSV disease. RSV is the leading cause of hospitalization among U.S. infants (*6*); 2% to 3% of young infants will be hospitalized for RSV disease (*7*–*9*). Approximately 58,000–80,000 RSV-associated hospitalizations and 100–300 RSV-associated deaths occur annually among U.S. children aged <5 years (*10*–*13*). An estimated 79% of children aged <2 years hospitalized with RSV had no underlying medical conditions (*7*). RSV-associated hospitalization rates are highest in infants aged <6 months, with hospitalization peaking at age 1 month, and then decreasing with increasing age (*7*).

Before the COVID-19 pandemic, RSV circulation consistently peaked during winter months in the continental United States, although the timing varied by geographic region (*14*); however, the COVID-19 pandemic disrupted RSV seasonality, with historically low RSV circulation during 2020–21 and early and prolonged circulation during 2021–22 (*14*). RSV circulation in 2022–23 began later than during the 2021–22 season but earlier than prepandemic seasons (*14*). RSV activity in August and September 2023 suggests that transmission patterns are returning to prepandemic seasonal RSV trends.^†^

## Methods

Since October 2021, the ACIP RSV Vaccines Pediatric/Maternal Work Group (the Work Group) has met at least monthly to review evidence regarding RSV epidemiology and safety, efficacy, and potential economic impact of pediatric and maternal RSV prevention products, including RSVpreF vaccine. A systematic literature search was completed to review evidence regarding the efficacy and safety of maternal RSVpreF vaccination during pregnancy. The Work Group determined a priori outcomes that were critical or important to vaccine policy decisions.^§^ Evidence of efficacy and safety were derived from multicountry trials that randomized pregnant persons to receive maternal RSVpreF vaccination or placebo during 24–36 weeks’ gestation: a phase 2b trial^¶^ with 581 pregnant persons (115 of whom received the phase 3 vaccine dose and formulation and 117 of whom received placebo) and a phase 3 trial** including 7,392 pregnant persons, randomized 1:1 to vaccine and placebo arms (*15*,*16*). The Work Group used the Grading of Recommendations, Assessment, Development, and Evaluation (GRADE) approach^††^ to assess the certainty of evidence for outcomes related to maternal RSVpreF vaccination during pregnancy, rated on a scale of very low to high certainty. The Work Group employed the Evidence to Recommendation (EtR) Framework^§§^ to guide its deliberations on recommendations for maternal RSVpreF vaccination during pregnancy and review of data on the public health problem, benefits and harms, value to the target population, acceptability to key stakeholders, feasibility, direct and indirect resource utilization, and equity.

## Vaccine Efficacy and Safety

In the Pfizer phase 2b and 3 trials, maternal RSVpreF vaccination was administered during 24–36 weeks’ gestation (*15*,*16*). For the GRADE assessment, data were included from phase 2b and 3 trials using the trial dosing interval of 24–36 weeks’ gestation. Using all available data from the trial dosing interval provided increased power to detect potential benefits and harms. Additional analyses of efficacy and safety outcomes from participants who received vaccine or placebo during the approved dosing interval of 32–36 weeks’ gestation were reviewed and are included as a supplement to the evidence included in GRADE (*2*,*9*). The details of the GRADE evidence profile and supporting evidence for the EtR Framework are available at https://www.cdc.gov/vaccines/acip/recs/grade/pfizer-RSVpreF-pregnant-people.html and

https://www.cdc.gov/vaccines/acip/recs/grade/pfizer-RSVpreF-pregnant-people-etr.html.

### Vaccine Efficacy

For the GRADE assessment of benefits, data on vaccine efficacy among infants from birth through 180 days of life were evaluated (*9**,**16*). Efficacy against medically attended RSV-associated LRTI was 51.3% among the full trial population (trial dosing interval of 24–36 weeks’ gestation) and 57.3% when maternal RSVpreF vaccination was given during the approved dosing interval (32–36 weeks’ gestation). Efficacy against hospitalization for RSV-associated LRTI was 56.8% during the full trial dosing interval and 48.2% during the approved dosing interval (Table 1).

**TABLE 1:** Effect estimates for the Pfizer maternal RSVpreF vaccine for the trial dosing interval and the approved dosing interval

Outcome	VE or RR (CI)*	
	Trial dosing interval (24–36 weeks’ gestation)^†^	Approved dosing interval (32–36 weeks’ gestation)^§^
**Benefits (efficacy against outcome), (VE) assessed at age 0–180 days**		
Medically attended RSV-associated LRTI in infants	51.3 (29.4 to 66.8)^¶^	57.3 (29.8 to 74.7)
Severe medically attended RSV-associated LRTI in infants**	69.4 (44.3 to 84.1)^¶^	76.5 (41.3 to 92.1)
Hospitalization for RSV-associated LRTI	56.8 (10.1 to 80.7)^††^	48.2 (–22.9 to 79.6)
Intensive care unit admission from RSV hospitalization in infants	42.9 (–124.8 to 87.7)	One event in the vaccine group Two events in the placebo group
Mechanical ventilation from RSV hospitalization in infants	100 (–9.1 to 100)	Zero events in the vaccine group Two events in the placebo group
All-cause medically attended LRTI in infants	2.5 (–17.9 to 19.4)^††^	7.3 (–15.7 to 25.7)
All-cause hospitalization for LRTI in infants	28.9 (–2.0 to 50.8)	34.7 (–18.8 to 64.9)
**Harms (RR)** ^§§^		
Serious adverse events in pregnant persons^¶¶^	1.06 (0.95 to 1.17)	1.02 (0.87 to 1.20)
Reactogenicity (grade 3 or higher systemic reactions) in pregnant persons***	0.97 (0.72 to 1.31)	0.98 (0.62 to 1.54)
Serious adverse events in infants^†††^	1.01 (0.91 to 1.11)	1.04 (0.90 to 1.20)
Preterm birth (<37 weeks’ gestational age)	1.20 (0.99 to 1.46)	1.15 (0.82 to 1.61)

**Abbreviations:** GRADE = Grading of Recommendations, Assessments, Development, and Evaluations; LRTI = lower respiratory tract infection; RR = relative risk; RSV = respiratory syncytial virus; VE = vaccine efficacy.

* 95% CI unless otherwise noted. When 95% CI not used, the CI was adjusted using the Bonferroni procedure, accounting for the primary endpoints’ results.

^†^ Vaccine efficacy was calculated as (1 – [P / (1 – P)]) x 100%, where P is the number of cases in the RSVpreF group divided by the total number of cases.

^§^ Vaccine efficacy was calculated as (1 − [hP / (1 − P)]) x 100%, where P is the number of cases in the RSVpreF group divided by the total number of cases and h is the ratio of number of participants at risk in the placebo group to the number of participants at risk in the RSVpreF group.

^¶^ 97.58% CI.

** Severe medically attended RSV-associated LRTI was a co-primary endpoint of the phase 3 clinical trial. This outcome was not included by CDC’s Advisory Committee on Immunization Practices RSV Vaccines Pediatric/Maternal Work Group as an a priori GRADE outcome critical or important to vaccine policy decision making.

^††^ 99.17% CI.

^§§^ Pooled RR estimates were independently calculated using counts of events and participants in the phase 3 trial interim analysis and phase 2b trial among those who received the phase 3 vaccine formulation.

^¶¶^ Serious adverse events in pregnant persons were collected through 6 months after delivery.

*** Up to 7 days after injection. When selecting the a priori harm outcomes, CDC’s Advisory Committee on Immunization Practices RSV Vaccines Pediatric/Maternal Work Group defined reactogenicity as both local and systemic reactions. These data only reflect systemic reactions.

^†††^ Serious adverse events in infants were collected through 6 months after delivery.

### Vaccine Safety

For the GRADE assessment of harms, results from the phase 2b and phase 3 trials were pooled^¶¶^ (*9**,**16*). The overall evidence certainty using GRADE criteria was rated as very low, driven by the uncertainty in the critical harm outcome of preterm birth (<37 weeks’ gestation).*** ACIP judged the benefits of maternal RSVpreF vaccination at 32–36 weeks’ gestation to outweigh the potential risks for preterm birth and hypertensive disorders of pregnancy.

The most common local and systemic adverse reactions were pain at the injection site, headache, muscle pain, and nausea.^†††^ Although not statistically significant, in the full trial population more preterm births and hypertensive disorders of pregnancy (including preeclampsia) were observed in persons administered the vaccine rather than the placebo, and more infants whose mothers received the vaccine had low birthweight ≤5.5 lbs (≤2,500 g) and neonatal jaundice compared with infants whose mothers received the placebo.^§§§^ Pregnant persons at increased risk for preterm delivery were excluded from the phase 2b and phase 3 trials. In the full trial population, preeclampsia occurred among 1.8% (95% CI = 1.4%–2.3%) of vaccine recipients and in 1.4% (95% CI = 1.1%–1.9%) of placebo recipients (*2*). Pregnancy-related serious adverse events overall (which include preeclampsia) occurred in 16.2% (95% CI = 15.1%–17.5%) of participants in the vaccine group and 15.2% (95% CI = 14.0%–16.4%) in the placebo group^¶¶¶^ (*2*).

The data reviewed by ACIP support that limiting vaccine administration to the approved dosing interval (32–36 weeks’ gestation) reduces the potential risk for preterm birth and thereby, the potential for related complications compared with the trial dosing interval of 24–36 weeks’ gestation. In the Pfizer phase 3 trial, using the full trial dosing interval, 5.7% of infants born to RSVpreF vaccine recipients were preterm compared with 4.7% of those born to placebo recipients (Table 2). In the full trial population, more than one half of preterm births occurred >30 days after vaccination (121 [60%] of 201 preterm births in the vaccine group and 98 [58%] of 169 preterm births in the placebo group), and most preterm births occurred at or after 33 weeks’ gestation (194 [97%] of 201 preterm births in the vaccine group versus 161 [95%] of 169 preterm births in the placebo group). When the prevalence of preterm birth was assessed among phase 3 trial participants who received vaccine during the approved dosing interval (32–36 weeks’ gestation), 4.2% of infants were born preterm in the vaccine group versus 3.7% in the placebo group. The majority of preterm births among participants who received vaccination during the approved dosing interval occurred at 36 weeks’ gestation (49 [72%] of 68 preterm births in the vaccine group and 35 [59%] of 59 preterm births in the placebo group).

**TABLE 2:** Preterm birth (<37 weeks’ gestation), low birthweight and neonatal jaundice outcomes in Pfizer RSVpreF vaccine phase 3 trial for the trial dosing interval and the approved dosing interval*

Outcome	Group, trial dosing interval (24–36 wks’ gestation)^†^				Group, approved dosing interval (32–36 wks’ gestation)^§^			
	RSVpreF N = 3,568		Placebo N = 3,558		RSVpreF N = 1,628		Placebo N = 1,604	
	No.	% (95% CI)	No.	% (95% CI)	No.	% (95% CI)	No.	% (95% CI)
**Preterm birth^¶^**	202	5.7 (4.9–6.5)	169	4.7 (4.1–5.5)	68	4.2 (3.3–5.3)	59	3.7 (2.8–4.7)
Low birthweight**	181	5.1 (4.4–5.8)	155	4.4 (3.7–5.1)	67	4.1 (3.2–5.2)	54	3.4 (2.5–4.4)
**Neonatal jaundice**	257	7.2 (6.4–8.1)	240	6.7 (5.9–7.6)	102	6.3 (5.1–7.6)	107	6.7 (5.5–8.0)

* All differences between vaccine group and placebo group were not statistically significant, as determined by nonoverlapping CIs.

^†^ https://www.fda.gov/media/168889/download?attachment

^§^ Data obtained directly from the sponsor during August, 2023.

^¶^ Less than 37 weeks’ gestation.

** Less than ≤5.5 lbs (2,500 g).

The Pfizer maternal RSVpreF vaccine is the same formulation and dose approved for use in adults aged ≥60 years. In clinical trials in adults aged ≥60 years for RSVpreF vaccine, three inflammatory neurologic events (two cases of Guillain-Barré syndrome, including one case of the Miller-Fisher variant, and one case of undifferentiated motor-sensory polyneuropathy) were reported within 42 days after vaccination among 20,255 investigational vaccine recipients aged ≥60 years, whereas no cases were observed among placebo recipients (*17*). No cases of Guillain-Barré syndrome or other inflammatory neurologic events were reported in the phase 2b or phase 3 trials among pregnant persons (*15*).

## Economic Analysis

ACIP considered whether use of RSVpreF vaccine in pregnant persons is a reasonable and efficient allocation of resources. The societal incremental cost effectiveness ratio for RSVpreF vaccine, assuming year-round dosing and cost of $295 per dose, was $400,304 per quality-adjusted life year (QALY) saved. Assuming a pre–COVID-19 typical RSV seasonality in most of the continental United States, the societal incremental cost effectiveness ratio for administering RSVpreF to pregnant persons during September–January would be $167,280/QALY saved (*9*).

## Recommendations for Use of RSVpreF Vaccine in Pregnant Persons

On September 22, 2023, ACIP and CDC recommended maternal Pfizer RSVpreF vaccination in pregnant persons as a one-time dose at 32 weeks and zero days’–36 weeks and 6 days’ gestation using seasonal administration (meaning September–January in most of the continental United States) for prevention of RSV-associated LRTI in infants aged <6 months.**** These recommendations will be updated as new evidence becomes available.

### Clinical Guidance

**Seasonal Administration of RSVpreF Vaccine**. Maternal RSVpreF vaccine should be administered to pregnant persons during September–January in most of the continental United States to target vaccine to pregnant persons whose infants will be in their first months of life, when protection from maternal vaccination would be at its highest, during the RSV season. Administering maternal RSVpreF vaccine starting in September (1–2 months before the anticipated start of the RSV season) and continuing through January (2–3 months before the anticipated end of the RSV season) will maximize cost-effectiveness and benefits. In jurisdictions with RSV seasonality that differs from most of the continental United States, including Alaska, southern Florida, Guam, Hawaii, Puerto Rico, U.S.-affiliated Pacific Islands, and U.S. Virgin Islands, providers should follow state, local, or territorial guidance on timing of maternal RSVpreF vaccination.^††††^

**Simultaneous Administration with Other Vaccines**. In accordance with CDC’s General Best Practices Guidelines for Immunization, maternal RSVpreF vaccine can be administered to pregnant persons with other recommended vaccines, such as tetanus, diphtheria, and pertussis (Tdap), influenza, and COVID-19 vaccines, without regard to timing, including simultaneous vaccination at different anatomic sites on the same day (*18*).

**Additional Vaccine Doses in Subsequent Pregnancies**. Currently, no data are available on either the efficacy of the first lifetime dose to protect infants born after subsequent pregnancies or the safety of additional doses given during subsequent pregnancies. Additional data are needed to determine whether additional seasonal doses during subsequent pregnancies are indicated, and ACIP might update recommendations in the future, as data become available.

### Updated Clinical Guidance for Use of Nirsevimab and Maternal RSVpreF Vaccine

Recommendations for nirsevimab, a long-acting monoclonal antibody product, have been previously published (*3*). Either maternal RSVpreF vaccination during pregnancy at 32–36 weeks’ gestation or nirsevimab immunization for infants aged <8 months who are born during or are entering their first RSV season is recommended to prevent RSV-associated LRTI in infants, but administration of both products is not needed for most infants. Providers who care for pregnant persons should discuss the relative advantages and disadvantages of both maternal RSVpreF vaccination and nirsevimab and consider patient preferences when determining whether to vaccinate the pregnant person or to rely on administration of nirsevimab to the infant (Box) (*19*).

BOX **Relative advantages and disadvantages of maternal RSVpreF vaccination and nirsevimab administration to infants to prevent respiratory syncytial virus lower respiratory tract infection in infants — United States, 2023**

**Maternal RSVpreF vaccination**

**Advantages**

• Provides protection immediately after birth

• Might be more resistant to potential mutations in F protein*

**Disadvantages**

• Protection potentially reduced if fewer antibodies are produced or are transferred from pregnant person to baby (e.g., pregnant person is immunocompromised or infant born soon after vaccination)

• Potential risk for preterm birth and hypertensive disorders of pregnancy

**Infant nirsevimab administration**

**Advantages**

• Studies of antibody levels suggest that protection might wane more slowly than protection from the maternal RSV vaccine

• Assures direct receipt of antibodies rather than relying on transplacental transfer

• No risk for adverse pregnancy outcomes

**Disadvantages**

• Potentially limited availability during 2023–24 RSV season

• Requires infant injection

**Abbreviation**: RSV = respiratory syncytial virus.

* Maternal RSV vaccination results in a polyclonal immune response, which is expected to be more resistant to potential mutations in the RSV F protein than a monoclonal antibody product.

No data are available directly comparing the efficacy of nirsevimab and maternal RSVpreF vaccine in preventing RSV-associated LRTI in infants. Protection conferred through maternal vaccination will likely wane after 3 months, as has been observed in infants born to pregnant persons who have received influenza and COVID-19 vaccines (*16*,*20*,*21*). However, because maternal RSV vaccination at 32–36 weeks’ gestation is recommended during only September–January in most of the continental United States, most infants of vaccinated mothers will be born during an RSV season. Mothers of most infants born outside of RSV season (i.e., during April–September) will not have been vaccinated; therefore, nirsevimab is recommended for these infants at the onset of the RSV season if they are aged <8 months.

At least 14 days are likely needed after maternal vaccination for development and transplacental transfer of maternal antibodies to protect the infant (*16*,*22*); therefore, nirsevimab is recommended for infants born <14 days after maternal RSVpreF vaccination. The earliest an infant could be born and be considered protected by maternal receipt of RSVpreF vaccine at 32 weeks’ gestation (the earliest recommended time for vaccination) would be at 34 gestational weeks. Therefore, nirsevimab is recommended for all infants born at <34 weeks’ gestation.

Nirsevimab is recommended for infants aged <8 months born during or entering their first RSV season whose mother did not receive RSVpreF vaccine, whose mother’s receipt of RSVpreF vaccine is unknown, or who were born <14 days after maternal vaccination. Nirsevimab is not needed for most infants aged <8 months whose mother received RSVpreF vaccine ≥14 days before birth. Nirsevimab may be considered for infants born to vaccinated mothers in rare circumstances when, based on the clinical judgment of the health care provider, the potential incremental benefit of administration is warranted. These situations include, but are not limited to, infants born to mothers who might not have mounted an adequate immune response to vaccination (e.g., persons with immunocompromising conditions) or who have conditions associated with reduced transplacental antibody transfer (e.g., persons living with HIV infection) (*23*); infants who might have experienced loss of maternal antibodies, such as those who have undergone cardiopulmonary bypass (*24*) or extracorporeal membrane oxygenation; and infants with substantially increased risk for severe RSV disease (e.g., hemodynamically significant congenital heart disease, or intensive care admission requiring oxygen at hospital discharge).

Infants and children aged 8–19 months who are at increased risk for severe RSV disease and are entering their second RSV season are recommended to receive nirsevimab regardless of maternal RSVpreF vaccination (*3*). Recommendations for timing of nirsevimab administration, coadministration of nirsevimab with routine childhood vaccines, reporting of adverse events, and recommendations for use for infants and children aged 8–19 months who are at increased risk for severe RSV disease and who are entering their second RSV season have been previously published and remain unchanged (*3*).

### Precautions and Contraindications

As with all vaccines, RSV vaccination should be delayed for persons experiencing moderate or severe acute illness with or without fever (precaution). RSV vaccines are contraindicated for and should not be administered to persons with a history of severe allergic reaction, such as anaphylaxis, to any component of the vaccine.

### Reporting of Vaccine Adverse Events

Adverse events after vaccination should be reported to the Vaccine Adverse Event Reporting System (VAERS). Reporting is encouraged for any clinically significant adverse event even if it is uncertain whether the vaccine caused the event. Information on how to submit a report to VAERS is available at https://vaers.hhs.gov/index.html or by telephone at 1-800-822-7967.

### Future Research and Monitoring Priorities

CDC will monitor adverse events, including preterm birth, hypertensive disorders of pregnancy, and inflammatory neurologic events after RSVpreF vaccination in pregnant persons through VAERS and the Vaccine Safety Datalink (https://www.cdc.gov/vaccinesafety/ensuringsafety/monitoring/vsd/index.html). Reactions and health impacts after RSVpreF vaccination will also be monitored through v-safe. According to FDA post-marketing requirements, the manufacturer will conduct post-marketing studies to assess preterm birth and hypertensive disorders of pregnancy, including preeclampsia (*25*).

## Footnotes

* These authors contributed equally to this report.

† https://emergency.cdc.gov/han/2023/han00498.asp; https://www.cdc.gov/surveillance/nrevss/rsv/index.html

§ Critical outcomes: medically attended RSV-associated LRTI in infants, hospitalization for RSV-associated LRTI in infants, serious adverse events in pregnant persons, serious adverse events in infants, and preterm birth (<37 weeks’ gestation). Important outcomes: intensive care unit (ICU) admission from RSV hospitalization in infants, mechanical ventilation from RSV hospitalization in infants, RSV-associated death in infants, all-cause medically attended LRTI in infants, all-cause hospitalization for LRTI in infants, and reactogenicity (grade 3 or higher) in pregnant persons.

¶ Trial conducted in Argentina, Chile, New Zealand, South Africa, and United States.

** Trial conducted in Argentina, Australia, Brazil, Canada, Chile, Denmark, Finland, The Gambia, Japan, Mexico, Netherlands, New Zealand, Philippines, South Africa, South Korea, Spain, Taiwan, and United States.

††
https://www.cdc.gov/vaccines/acip/recs/grade/about-grade.html

§§
https://www.cdc.gov/vaccines/acip/recs/grade/downloads/acip-evidence-recs-framework.pdf

¶¶ A serious adverse event is defined as any untoward medical occurrence that results in death, is life-threatening, requires inpatient hospitalization or prolongation of existing hospitalization, results in persistent disability or incapacity, or is a congenital anomaly or birth defect. Serious adverse events in pregnant persons were collected ≤6 months after delivery. Serious adverse events in infants were collected ≤12 months after delivery. Reactogenicity events were collected ≤7 days following injection.

*** The outcome of preterm birth was rated as very low certainty. Very serious concern for imprecision was noted because of the CI range containing estimates for which different policy decisions might be considered as well as not meeting optimum information requirements. In addition, serious concern for indirectness was present as 55% of participants in the phase 3 trial and 62% of participants in the phase 2b trial did not receive vaccine or placebo in the approved dosing interval (32–36 weeks’ gestation). In the approved dosing interval, there is less opportunity for serious adverse events, including preterm birth, compared with the trial dosing interval (24–36 weeks’ gestation).

††† In the phase 3 trial among 3,663 RSVpreF recipients and 3,638 to 3,639 placebo recipients, injection site pain was reported by 40.6% of RSVpreF and 10.1% of placebo recipients; headache by 31.0% of RSVpreF and 27.6% of placebo recipients; muscle pain by 26.5% of RSVpreF and 17.1% placebo recipients; and nausea by 20.0% of RSVpreF and 19.2% of placebo recipients.

§§§ Low birthweight ≤5.5 lbs (≤2,500 g) and neonatal jaundice are more common among infants born preterm than among infants born at term. https://www.marchofdimes.org/find-support/topics/birth/premature-babies

¶¶¶ Among the full trial population, gestational hypertension occurred in 1.1% (95% CI = 0.8%–1.5%) of vaccine recipients and 1.0% (95% CI = 0.7%–1.4%) of placebo recipients. Hypertension occurred in 0.4% (95% CI = 0.2%–0.6%) of vaccine recipients and 0.2% (95% CI = 0.1%–0.4%) of placebo recipients.

**** On September 22, 2023, ACIP voted 11–1 in favor of the recommendation: maternal RSV vaccine is recommended for pregnant persons during 32–36 weeks’ gestation, using seasonal administration, to prevent RSV-associated LRTI in infants.

†††† The timing of maternal RSVpreF vaccination might vary in these jurisdictions because the historic timing of RSV circulation differs from the rest of the United States. As maternal RSVpreF vaccination should start 1–2 months before the anticipated start of the RSV season and continue through 2–3 months before the anticipated end of the RSV season, it is not feasible to change maternal RSVpreF vaccination timing based on year-to-year variations in RSV circulation. Thus, in most of the continental United States, maternal RSVpreF vaccination should be given in September–January, regardless of year-to-year variation in RSV circulation.
